# A historical overview of legislated alcohol policy in the Northern Territory of Australia: 1979–2021

**DOI:** 10.1186/s12889-021-11957-5

**Published:** 2021-10-23

**Authors:** Sarah Clifford, James A. Smith, Michael Livingston, Cassandra J. C. Wright, Kalinda E. Griffiths, Peter G. Miller

**Affiliations:** 1 Menzies School of Health Research, Charles Darwin University, Darwin, NT Australia; 2 Centre for Drug use, Addictive and Anti-social behaviour Research (CEDAAR), Deakin University, Geelong, VIC Australia; 3 Centre for Alcohol Policy Research, La Trobe University, Melbourne, VIC Australia; 4 National Drug Research Institute, Curtin University, Melbourne, VIC Australia; 5 Centre for Big Data Research in Health, University of New South Wales, Sydney, NSW Australia; 6 Melbourne School of Population and Global Health, University of Melbourne, Melbourne, Australia

**Keywords:** Alcohol policy, Alcohol legislation, Northern Territory, Evaluation

## Abstract

**Background:**

The Northern Territory (NT) has the highest levels of alcohol consumption and harms in Australia. Since the creation of the NT Liquor Act 1978, which came into effect in 1979, numerous legislated alcohol policies have been introduced to attempt to address these harms. We present a narrative historical overview of alcohol policies implemented in the NT from 1979 to 2021.

**Methods:**

Using scoping review methodology, databases were searched from 1979 to 2021. Of 506 articles screened, 34 met inclusion criteria. Reference lists of all included articles were searched, resulting in the inclusion of another 41 articles and reports, totalling 75 final documents. Policies were organised using Babor and colleagues (2010) established framework: 1. pricing/ taxation; 2. regulating physical availability; 3. modifying drinking environments; 4. drink-driving countermeasures; 5. restrictions on marketing; 6. education/persuasion; 7. treatment/early intervention.

**Results:**

Two pricing/taxation policies have been implemented, Living With Alcohol (LWA) and Minimum Unit Price, both demonstrating evidence of positive effects on health and consumption outcomes. Eight policies approaches have focused on regulating physical availability, implemented at both individual and local area levels. Several of these policies have varied by location and been amended over time. There is some evidence demonstrating reduction in harms attributable to Liquor Supply Plans, localised restrictions, and General Restricted Areas, although these have been site specific. Of the three policies which targeted modifying the drinking environment; one was evaluated, finding a relocation of social harms, rather than a reduction. The literature outlines a range of controversies, particularly regarding policies in domain 2–3, including racial discrimination and a lack of policy stability. No policies relating to restricting marketing or education/persuasion programs were found. The only drink-driving legislated policy was considered to have contributed to the success of the LWA program. Three policies relating to treatment were described; two were not evaluated and evidence showed no ongoing benefits of Alcohol Mandatory Treatment.

**Discussion:**

The NT has implemented a large number of alcohol policies, several of which have evidence of positive effects. However, these policies have often existed in a context of clear politicisation of alcohol policy, frequently with an implicit focus on Aboriginal people’s consumption.

**Supplementary Information:**

The online version contains supplementary material available at 10.1186/s12889-021-11957-5.

## Introduction

**Fig. 1:**
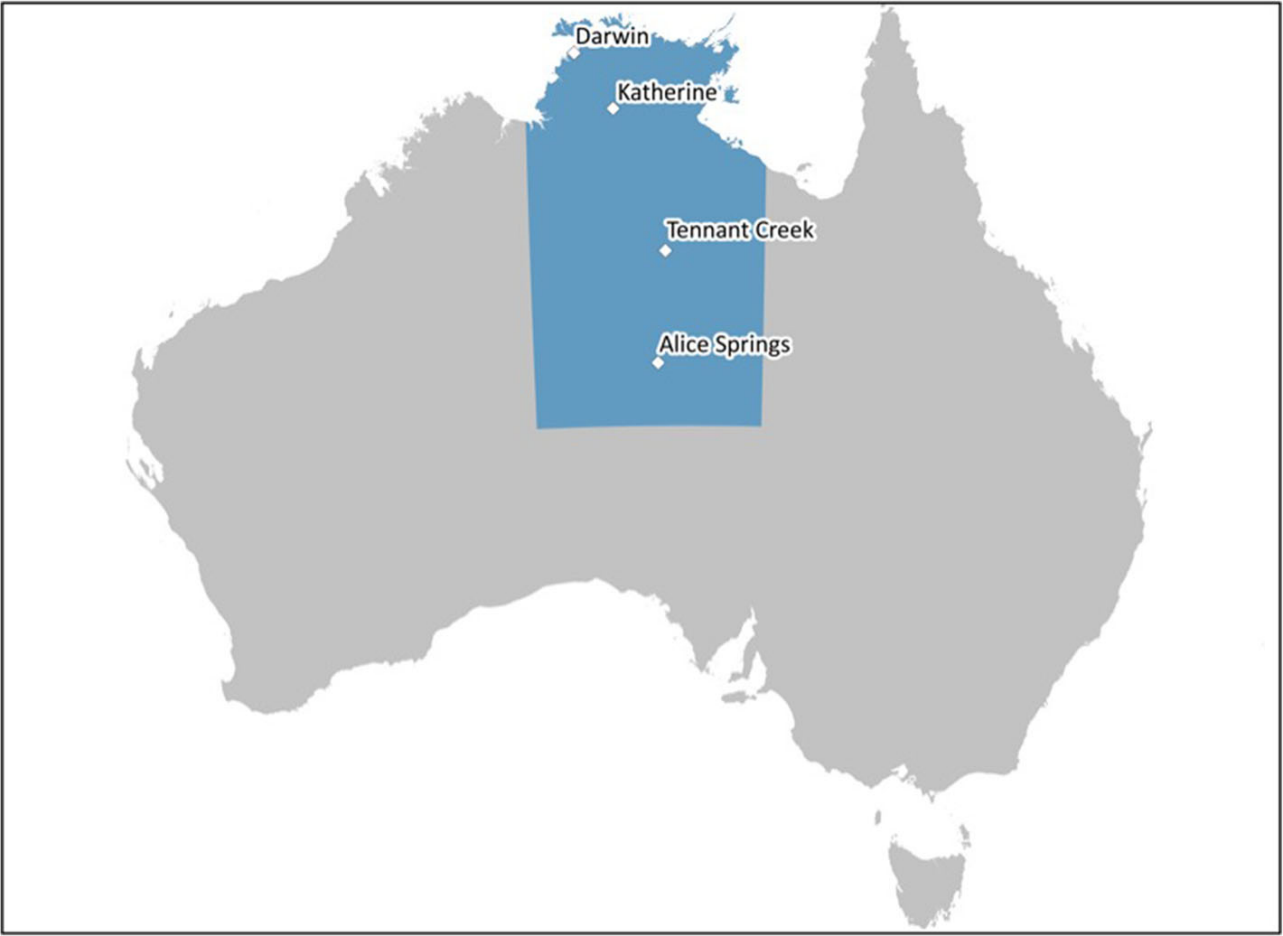
Map of the Northern Territory

Alcohol is one of the leading preventable causes of death and disability worldwide [1] and its harmful use is especially problematic within the Northern Territory of Australia (NT) [2]. Since at least the 1980s the NT’s per capita consumption has well exceeded the national average [3] and the NT continues to have a greater proportion of adult residents who exceed both alcohol consumption lifetime risk (21.4%) and single occasion risk (49.1%) compared to the national averages (16.1 and 42.1%, respectively) [4]. The NT is the least populous of all of Australian jurisdictions [5] and has notably different population demographics. The NT population is younger, with a slightly higher proportion of male residents, and tenfold the proportion of Aboriginal and Torres Strait Islander^1^ residents compared to the national population [6]. Darwin is the capital city, and 60% of Territorians reside in the Greater Darwin region [5]. Other notable population centres include Alice Springs (accounting for 10.7% of the NT population), Katherine (4.3%) and Tennant Creek (1.3%) (see Fig. 1) [5].

As the name suggests, the NT a federal territory, not a state. It has only been a self-governing territory since 1978; prior to this the NT was managed (formally called administered) by the Commonwealth. Self-governance was swiftly followed by the implementation of the *Liquor Act (NT) 1978*, which came into effect on 12 February 1979. In the more than four decades since, there have been a slew of legislative approaches which have attempted to reduce alcohol consumption and related harms. Alcohol policy in the NT has been heavily contested, with alcohol policies frequently a prominent feature of political party’s election campaigns. Both major political parties, the Country Liberal Party and the Australian Labor Party have received donations from the alcohol industry [7, 8].

### The current policy context in the Northern Territory

The reintroduction of a Banned Drinker Register (BDR2) was part of the 2016 NT Australian Labor Party’s election platform. The BDR, as the name would suggest, is a register of individuals who are banned from purchasing alcohol. The BDR is enforced at takeaway outlets by scanning identification at point of sale [9]. Enacted in September 2017; the BDR2 was soon followed by the formalisation of police monitoring of takeaway alcohol outlets (a practise which has been ongoing since 2012) through the establishment of Police Auxiliary Liquor Inspectors (PALIs). PALIs are uniformed inspectors, stationed at takeaway alcohol outlets, who seek to prevent the consumption of alcohol in public and restricted areas. PALIs request a form of identification and query customers regarding their intended drinking location. Purchase will be prevented if a valid (non-restricted) address cannot be provided [10]. The first cohort of PALIs graduated in August 2018 and on 1st October 2018 a $1.30 Minimum Unit Price (MUP) on alcohol was introduced. The latter reforms were driven by the Northern Territory Alcohol Policies and Legislation Review [11] commissioned by the newly elected Labor Government. This is the first implementation of a MUP in Australia, and both the BDR and PALIs are currently unique to the NT. Each of these policies have received significant investment and been enshrined in corresponding legislation (henceforth termed legislated alcohol policy).

### The purpose of this historical overview

Ongoing, robust evaluation of these policies is essential [12], and an understanding of the relevant historical context, particularly previous policy evaluations, can help to inform approaches to future evaluations. Babor and colleagues [13] define alcohol policy “as any purposeful effort or authoritative decision on the part of governments to minimize or prevent alcohol-related consequences”. Both Babor et al. [13] and Ritter & Stoove [14] further highlight that alcohol policies include both specific strategies (such as taxation) and the allocation of resources to prevention and treatment. The allocation of resources, however, are generally not underpinned by legislation (Alcohol Mandatory Treatment is a notable exception). This paper is specifically interested in legislated alcohol policy and as such, is not a complete overview of all NT alcohol policies. The reason for this is twofold. Firstly, there are significant challenges associated with mapping historical unlegislated policies because they are less likely to be evaluated and therefore less likely to have been captured in academic or grey literature. Secondly, it is likely that the volume of all alcohol policies over more than forty years would prove unmanageable to describe in a digestible way. We posit that there is value in a focused examination of legislated policies, to allow for greater depth and consideration. For the purpose of this paper we have considered both legislation; that is the law itself as an Act passed through Parliament, which can only be amended through another Act of Parliament; and regulations, which are the guidelines that dictate how provisions of an Act are applied [15]. We hope this paper will provide a reference point for those examining alcohol policy in the NT, allowing them to understand what has been done previously and located relevant evidence related to the policies of interest.

## Methods

This overview was undertaken using scoping review methodology [16]. PRISMA (preferred reporting items for systematic reviews and meta-analyses) protocol [17] was used to identify literature. An a-priori electronic database search strategy was employed: specific databases included were Scopus, PubMed, SAGE Journals, PSYCInfo, and the NT Department of Health (DoH) Publications Collection.

### Search strategy

Table 1 outlines the search of five electronic databases for primary academic research, reports and commentaries. The search terms included were: alcohol; policy; intervention; program; ‘Northern Territory’; ‘Top End’ and ‘Central Australia’ (located within the abstract) for Scopus, PubMed, SAGE Journals and PsycINFO. Northern Territory Department of Health (DoH) Publications Collection was searched with just the term ‘alcohol’. Year of publication was limited in all searches from 1979 onwards.

**Table 1:** Databases, search terms, and records returned [as of 24 May 2021]

Order of search	Electronic database	Range of disciplines	Search terms	No. of records returned	No. of records retained
1	PubMed	Medicine, nursing, toxicology, nutrition, life sciences, and more	(alcohol[Title/Abstract]) AND (policy[Title/Abstract] OR intervention[Title/Abstract] OR program[Title/Abstract])) AND (“Northern Territory”[Title/Abstract] OR “central Australia”[Title/Abstract] OR “Top End”[Title/Abstract])	43	16 (26 did not meet inclusion criteria; 1 duplicate)
2	PsycINFO	Psychology and related disciplines (e.g., medicine, neuroscience, and nursing)	AB alcohol AND AB (policy OR intervention OR program) AND AB “northern territory” OR AB “top end” OR AB “central Australia”	139	4 (124 did not meet inclusion criteria; 11 duplicates)
3	Scopus	Chemical and biological sciences, medical and health sciences, physical sciences, psychology, law, economics, human society, education and policy	ABS (alcohol) AND ABS (policy OR program OR intervention) AND ABS (“Northern Territory”) OR ABS (“Top End”) OR ABS (“Central Australia”)	66	4 (53 did not meet inclusion criteria; 11 duplicates)
4	SAGE Journals	450 journal titles in business, humanities, social sciences and science, technology and medicine	[Abstract alcohol] AND [Abstract policy] AND [Abstract “northern territory”]	8	1 (3 did not meet inclusion criteria; 4 duplicates)
5	NT DoH Publications Collection	A digital repository for managing and storing publications produced by NT DoH	Alcohol	250	9; one record included 2 reports totalling 10 documents (236 did not meet inclusion criteria; 5 duplicates)

### Eligibility criteria

Publications were included if they evaluated or commented on one or more legislated alcohol policy in the NT and were academic primary research, academic commentary, or a government or non-government evaluation report published in the English language between 1979 and 2021. Publications were excluded if they solely examined alcohol use or harms in the NT without addressing a legislated alcohol policy; solely presented legislated alcohol policy-related data (e.g. the number of people referred into Alcohol Mandatory Treatment each quarter) without accompanying evaluation or comment; recommended or preceded the alcohol-focused legislative intervention in question; were published policy documents, legislation (Acts) and regulations, media reports, and/or submissions to reviews or inquiries; evaluated or commented on alcohol policy that was not underpinned by legislation; and/or evaluated or commented on social policies which were likely to impact alcohol consumption or harms (e.g., community policing, managed welfare). As Liquor Supply Plans, a legislated policy [18], were evaluated concurrently with NT Alcohol Management Plans, an unlegislated policy, in Alice Springs [18], Tennant Creek [19] and Katherine [20] both have been captured within this overview.

**Fig. 2:**
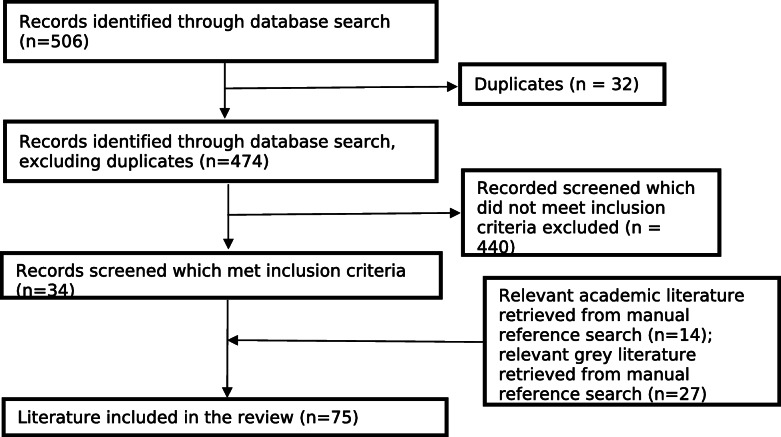
Flow diagram of literature inclusion for NT legislated alcohol policy overview

Reference lists of both grey and academic literature were then manually searched for other relevant articles, resulting in an additional 14 academic articles and 27 grey literature documents (Fig. 2). Several articles and reports identified through reference list searches were not available online and, in these instances, SC sourced them from a listed author. As this paper is not a systematic review, it was considered that one reviewer was sufficient. Literature which met the inclusion criteria was then reviewed and the alcohol policies identified within these documents were categorised using Babor et al.’s [13] seven policy areas (see Table 3). The seven policy areas are: 1. pricing and taxation; 2. regulating physical availability; 3. modifying the drinking environment; 4. drink-driving countermeasures; 5. restrictions on marketing; 6. education and persuasion; and 7. treatment and early intervention. In 2007 the National Drug Research Institute conducted a review of evidence and outcomes for restrictions of sale and supply of alcohol, including those enacted in the NT. To avoid duplication of this work, we have presented a descriptive overview of evaluation outcomes from 2007 onwards in the Supplementary Material. For those predating 2007, please refer to [18].

## Results

Over the past forty years there has been an increasing volume of work published relating to alcohol focused legislative interventions in the NT (Table 2).

**Table 2:** Descriptive overview of NT legislated alcohol policy documents identified

	Groups	No. of articles reporting	% articles reporting
Total number of documents		75	100%
Year of publication	1979–1990	9	12%
	1991–2000	17	23%
	2001–2010	19	25%
	2011 - May 2021	30	40%
Type of publication	Evaluation report	25	33%
	Primary research article (including those derived from evaluation reports)	20	27%
	Academic commentary	14	19%
	Government report	9	12%
	Other (i.e. conference presentation, other reports, synthesise of evidence, editorial)	7	9%

Most interventions have been addressed in several documents, for example: a grey literature evaluation report, a related academic publication, and/or an academic commentary. All identified policies and the related documents are outlined in Table 3.

**Table 3:** Northern Territory alcohol-focused legislative interventions from 1979 to 2020 and respective publications arising

Policy	Active years	Legislation	Location	Relevant publications and authors	Official evaluation	Babor’s area of alcohol policy
General Restricted Areas (including dry communities, remote community clubs and permit systems)	12 February 1979 – present	Originally: *Liquor Act 1978 (NT) Section 81* Present: *Liquor Act 2019 (NT) Section 172–187*	In theory could be requested by any community In practise has only requested by Aboriginal communities	Overall GRA evaluations: d’Abbs [21]; Northern Territory Liquor Commission [22] Inquiry: Sessional Committee on Use and Abuse of Alcohol by the Community [23] Jaburi club sale restrictions evaluation: d’Abbs & Togni [24] + related academic publication: d’Abbs [25] Groote Eylandt permit scheme evaluation: Conigrave et al. [26] NT-wide evaluations of clubs: Shaw et al. [27] Gove Penisula permit scheme evaluation: d’Abbs et al. [28] NT-wide permit schemes evaluation report: d’Abbs & Crundall [29] +related academic publication: d’Abbs & Crundall [18] Policy analysis: d’Abbs et al. [19]; d’Abbs et al. [20] Discussed in: Barazani [30]; d’Abbs [31–34]; Larkins & McDonald [35]; NDRI [36]	Yes	2. Regulating physical availability
Change to NT wide takeaway alcohol outlet trading hours	July 1982 – unknown	*Liquor (Amendment) Regulations 1982 No. 4 (NT)*	NT wide	Evaluation: Drug and Alcohol Bureau [37]	Yes	2. Regulating physical availability
Public Drinking Legislation (2 km Law) (now included under Prohibited Public Places)	1982 – present	Originally: *Summary Offences Act (NT) Section 45D* Shifted to *Liquor Act 1978 (NT) Part VIIIB* in 2012 Present: *Liquor Act 2019 (NT) Section 171*	NT wide	Evaluations: Drug and Alcohol Bureau [38]; O’Connor [39] +related academic publication: O’Connor [40] Discussed in: Barazani [30]; d’Abbs [31, 32, 34, 41]; Larkins & McDonald [35]; National Drug Research Institute [36]	Yes	3. Modifying the drinking environment
Regulating strip shows in public bars	1989	*Liquor Amendment Act 1989 (NT)*	NT-wide	Academic publication: Boffa et al. [42]	No	3. Modifying the drinking environment
Living with Alcohol Program	1 April 1992 – Dec 2002 Excise tax ceased 5 August 1997	Originally: *Amendments of the Liquor Regulations 1992 (NT)* Impacted by: High Court of Australia combined decision in the cases of *Walter Hammond and Associates v the State of NSW and others* and *Ha and anor v the State of NSW and others*	NT wide	Overall evaluations: Crundall [43]; Chikritzhs et al. [3]; Chikritzhs et al. [44] + related academic publications: Chikritzhs et al. [45]; Stockwell et al. [46] Coin-operated breathalysers evaluation: Crundall [47] Cask Wine Levy evaluation (academic publication): Gray et al. [48] Commentaries: d’Abbs [49], d’Abbs [50]; Holder [51] (1 x response to Holder; Chikritzhs, Stockwell & Pascal [52]) Discussed in: Barazani [30]	Yes	1. Pricing and taxation 2. Regulating physical availability 6. Education and persuasion 7. Treatment and early intervention
Elliott Restrictions	1993 – unknown	Originally*: Liquor Act 1978 (NT) Section 33* Now: *Liquor Act 2019 (NT) Section 113–115*	Elliott	Evaluation: Bennett et al. [53] Academic publication: Walley & Trindall [54] Discussed in: d’Abbs & Togni [20]; NDRI [36]	Yes	2. Regulating physical availability
Lowing BAC	Dec 1994 - present	*Traffic Amendment Act 1994 (NT)*	NT-wide	Discussed in d’Abbs [50]; Stockwell et al. [46]	No	4. Drink-driving prevention and countermeasures
Racial Discrimination Act and ‘Special Measures’	1996 – present	*Racial Discrimination Act 1975 (Cwt) Section 8(1)*	Only applicable to Aboriginal communities	Evaluation (relevant to NT): d’Abbs et al. [55] Discussed in: Barazani [30]; d’Abbs [33]; d’Abbs & Togni [20]; Hunyor [56]; NDRI [36]	Yes	2. Regulating physical availability
Tennant Creek trial restrictions Ongoing restrictions Liquor Supply Plan / Alcohol Management Plan Emergency Restrictions	Aug 1995 – Feb 1996 20th April 1996 July 2006 – revised 1 August 2008 (Alcohol Management Plan added)- unknown 28 Feb 2018 - present	Originally: *Liquor Act 1978 (NT) Section 33* Present: *Liquor Act 2019 (NT) Section 113–115*	Tennant Creek	Evaluations: d’Abbs et al. [57], d’Abbs et al. [58]; d’Abbs et al. [59]; Gray et al. [60] +related academic publication: Gray et al. [61] Discussed in: Barazani [30], d’Abbs [33], d’Abbs & Togni [20], Ellis [62], NDRI [36], Smith et al. [63]	Yes	2. Regulating physical availability, 3. Modifying the drinking environment
Katherine restricted takeaway trading hours Liquor Supply Plan / Alcohol Management Plan	March 1999 - unknown 21 Jan 2007 – revised 2013 – unknown	Originally: *Liquor Act 1978 (NT) Section 33* Present: *Liquor Act 2019 (NT) Section 113–115.*	Katherine	Evaluations: d’Abbs et al. [64]; d’Abbs & Whitty [65] Discussed in: National Drug Research Institute [36]	Yes	2. Regulating physical availability
Alice Springs trial restrictions Liquor Supply Plan / Alcohol Management Plan	1 April 2002–31 March 2003 7 Sept 2006 – present with modifications	Originally: *Liquor Act 1978 (NT) Section 33* Present: *Liquor Act 2019 (NT) Section 113–115*	Alice Springs	Evaluations: Crundall & Moon [66] (responses Crundall [67]; Gray [68]); Senior et al. [69] (response MacKeith et al. [70]) Report: Symons et al. [71] Academic publication: Hogan et al. [72] Discussed in: Room [73]	Yes	2. Regulating physical availability, 3. Modifying the drinking environment
Alcohol Courts	8 March 2006 - unknown	Originally: *Alcohol Court Act 2006*	NT-wide	Discussed in: Senior et al. [69], MacKeith et al. [70], Symons et al. [71]	No	7. Treatment and early intervention
Public Restricted Areas (now included under Prohibited Public Places)	September 2006 – present	Originally*: Liquor Act 1978 (NT) Section 86A-86G* Present: *Liquor Act 2019 (NT) Section 171*	NT-wide	Discussed in: d’Abbs [41]; d’Abbs [33]; d’Abbs et al. [64]; MacKeith et al. (2009); NDRI (2007); Senior et al. (2009); Symons et al. (2012)	No	3. Modifying the drinking environment
Substance Misuse and Referral for Treatment (SMART) Courts	1 July 2011–1 July 2013	Originally: *Alcohol Reform (Substance Misuse and Referral for Treatment Court) Act 2011 (NT)* Repealed by: *Alcohol Mandatory Treatment Act 2013 (NT)*	NT wide	Discussed in: Buckley [74]	No	7. Treatment and early intervention
Banned Drinker Register v1	July 2011 – August 2012 (officially repealed 1 July 2013)	Originally: *Alcohol Reform (Prevention of Alcohol-related Crime and Substance Misuse) Act 2011* Repealed by: *Alcohol Mandatory Treatment Act 2013 (NT)*	NT wide	Discussed in: Buckley [74]; d’Abbs [41]; Room [73], Smith [75]; Smith & Adamson [76]	No	2. Regulating physical availability
Alcohol Mandatory Treatment	1 July 2013 – August 2016 (officially repealed 1 Sept 2017)	Originally: *Alcohol Mandatory Treatment Act 2013 (NT)* Repealed: *Alcohol Harm Reduction Act 2017 (NT) Section 46*	NT wide	Evaluation: PwC’s Indigenous Consulting [77] Academic publications: Lander et al. [78]; Ransley & Marchetti [79] Discussed in: Buckley [74]; d’Abbs [34]	Yes	7. Treatment and early intervention
Alcohol Protection Orders	Dec 2013–1 Sept 2017	Originally: *Alcohol Protection Orders Act 2013 (NT)* Repealed: *Alcohol Harm Reduction Act 2017 (NT) Section 46*	NT wide	Discussed in: Buckley [74]; d’Abbs [34]; Smith et al. [80]	No	2. Regulating physical availability
Banned Drinker Register v2	2 Sept 2017 – present	Originally: *Alcohol Harm Reduction Act 2017 (NT)* Present: *Liquor Act (NT) 2019, Section 128–131* and *Alcohol Harm Reduction Act 2017 (NT)*	NT wide	Evaluations: Ernst & Young [81]; Smith & Adamson [75]; Smith [76]; Smith et al. [80] Discussed in: Foundation for Alcohol Research and Education & People’s Alcohol Action Coalition [82]; Secombe et al. [83]; Smith et al. [12]; Wright, McAnulty & Secombe [84]	Yes	2. Regulating physical availability; 4. Drink driving countermeasures,
Police Auxiliary Liquor Inspectors (PALIs)	Aug 2018 – present	Originally: *Liquor Amendment (Point of Sale Intervention) Act 2018 (NT)* Present: *Liquor Act 2019 (NT) Section 249*	Katherine, Tennant Creek, Alice Springs	Discussed in: Clifford et al. [10]; Coomber et al. [85]; Foundation for Alcohol Research and Education & People’s Alcohol Action Coalition [82]; Secombe et al. [83]; Taylor et al. [86]; Wright, McAnulty & Secombe [84];	No	2. Regulating physical availability
Minimum Unit Price	1 Oct 2018 – present	Originally: *Liquor Amendment (Minimum Pricing) Act 2018 (NT)* Present: *Liquor Act 2019 (NT) Section 121*	NT wide	Evaluation: Coomber et al. [85] + related academic publication: Taylor et al. [86] Report: Foundation for Alcohol Research and Education & People’s Alcohol Action Coalition [82] Academic publications: Secombe et al. [87] (response: Clifford, Griffiths & Smith [10] Discussed in: Chikritzhs & Weeramanthri [88]; Secombe et al. [83]; Smith et al. [12]; Wright, McAnulty & Secombe [84]	Yes	1. Pricing and taxation

**Fig. 3:**
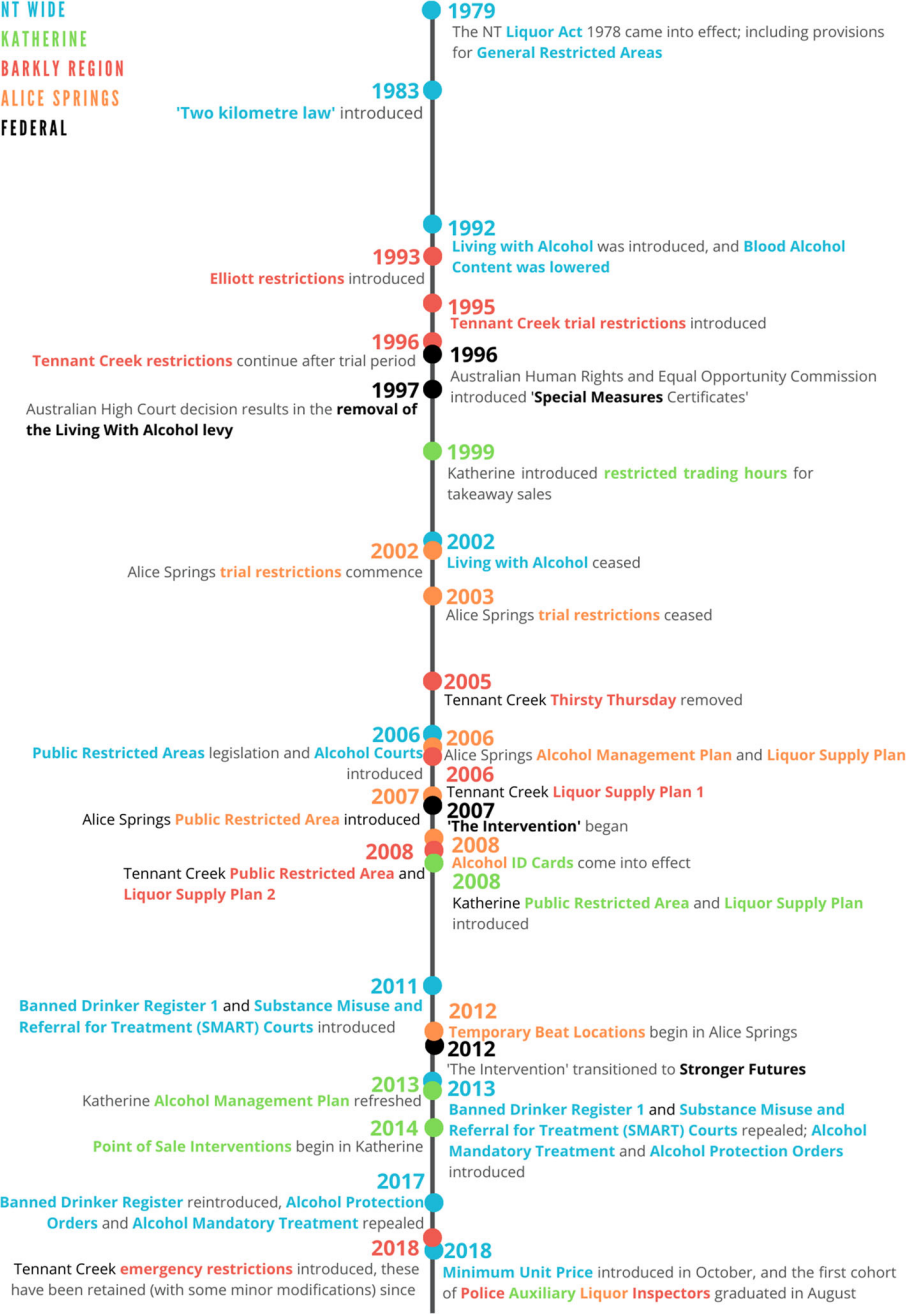
A timeline of alcohol-focused legislative interventions in the NT

Additionally, using the information contained within the identified literature, a timeline of NT and relevant federal interventions has been created for clarity (Fig. 3).

To provide additional context, the political party responsible for each intervention was identified.

### Pricing and taxation

In the addition to federal alcohol taxes, the NT has legisated pricing policies on two occasions.

### Living with alcohol (LWA) program

Implemented in 1992 by a Country Liberal majority government, LWA was a comprehensive population-wide harm minimisation program, funded through an excise tax on all beverages containing more than 3% alcohol [46]. In 1995, an additional levy of 35 cents per litre was added to cask wine. This was a hypothecated (‘earmarked’) tax, where the revenue was reinvested in harm minimisation activities including mass media education programs and local community initiatives such as night patrols and youth diversion and sports programs. Therefore, while LWA has been categorised under Pricing and Taxation it also involved regulating physical availability, education and persuasion, and treatment and early intervention. Both levies were removed on 5 August 1997 when a Federal High Court decision found the collection of a tobacco (or alcohol or petrol) excise tax by states and territories to be unconstitutional. Despite this, LWA did not formally cease until Dec 2002. LWA was comprehensively evaluated both pre [3, 46] and post [44, 45] the removal of the LWA levy. The additional cask wine levy was also evaluated in its own right [48]. The evaluation of the first four years found reductions in: acute and chronic alcohol-related death, per capita consumption, percentage of males drinking hazardously, and road trauma hospitalisations, although LWA was at least partly confounded by other interventions (e.g. the lowered BAC, see below) [46]. The cask wine levy was specifically shown to significantly reduce consumption [48]. Although after the 1997 High Court decision, the Commonwealth Government agreed to collect an equivalent amount and return it to the NT, it was no longer held in the LWA Trust Account and therefore was no longer ‘ringfenced’ for LWA [50]. An evaluation of 10 years of LWA demonstrated that in the absence of the levy, LWA did not show any evidence of a reduction in acute alcohol-related deaths. There was, however, a decrease in chronic alcohol-related deaths towards the end of the study period, potentially as a belated result of the reduced consumption brought about by the levy [45]. Chikritzhs et al. [45] concluded that there was a strong argument to combine alcohol taxes with comprehensive harm minimisation programs and services.

### Minimum unit price (MUP)

Nearly two decades later, on 1 October 2018, a Labor majority government introduced the first (and only) MUP in Australia. The MUP means that alcohol must be sold at a price above AU$1.30 per standard drink (10 g of ethanol). One year post implementation, Taylor and colleagues found immediate substantial declines in estimated cask wine consumption, and significant step reductions in total wine consumption [86]. There were also decreases in some alcohol-related harms, with variations between regions [85]. This regional variation was attributed to intersections with region-specific policies like PALIs (see below) [85]. Intensive care admissions have been examined in three academic articles [83, 84, 87], two focused on Alice Spring Hospital and one examining both Royal Darwin Hospital and Alice Springs Hospital. All have demonstrated reductions in the number of alcohol-related admissions. While the initial article [87] suggested that this could be attributed solely to MUP, subsequent outputs have acknowledged the impacts of other concurrently implemented regional alcohol policies.

### Regulating physical availability

For clarity, we present these policies categorised as impacting either Aboriginal communities; towns; or individuals.

#### Aboriginal community level

##### General restricted areas (GRAs)

The Liquor Act 1979 granted any community had the power to request the banning or restriction of alcohol in designated areas. These ‘restricted area provisions’, renamed GRAs in 2006, were rapidly taken up by Aboriginal communities. In 1986, 50 Aboriginal communities had GRAs [31], and by 2007 this number had risen to over 100 [36]. These requests, once approved by the Northern Territory Liquor Commission (NTLC),^2^ became law and therefore enforceable by police. In principle, these restrictions could take any form. In practice most communities had one or more of the following: a total ban; a ban of particular types of alcohol (often wine and spirits) with restricted access to others; a permit system under which specified individuals may drink in a community; or a licensed club, within which residents may drink subject to regulations (for example, mid-strength beer cans only, with a limit on the amount of cans per person per occasion) [31]. d’Abbs [31] specifies that the strength of these restrictions lie in the intersections of community control, to request and specify the form of restrictions, and statutory control, to subsequently enforce them (what he calls a complementary control model). There have been two evaluations of, and one inquiry into, these provisions and while some shortcomings were identified, overall a net benefit was identified [21–23]. Associated mechanisms, such as local clubs [24, 25, 27] and permit systems [18, 26, 29], have also been evaluated in reports and corresponding academic publications. The Curtin Springs Roadhouse represents an interesting case of an attempted GRA which eventually involved a Commonwealth organisation; the Australian Human Rights and Equal Opportunity Commission. In 1988 Curtin Springs Roadhouse began selling alcohol to Anangu.^3^ This catalysed nearly a decade of advocacy from the Ngaanyatjarra Pitjantjatjara Yangkunytjatjara Women’s Council who wished to restrict sales to members of their community. The NTLC was reticent to ratify a variation to the license; concerned about discrimination. The 1995 ‘Alcohol Report’ (Race Discrimination, Human Rights and the Distribution of Alcohol) made the case that prohibition of alcohol sales to Aboriginal people could be considered a special measure under the *Racial Discrimination Act (Cwt) 1975*. Since 1996 the Race Discrimination Commissioner has provided an opinion on whether a matter warrants a ‘special measure certificate’ [56]. These certificates are not legally binding but recognise that involved parties have acted in good faith [56]. The Curtin Springs Roadhouse certificate is the most notable NT example, and has been in effect since 1997 [55]. It is important to highlight that every community has had a different history of GRAs, and these have continued to evolve over time.

##### NT National Emergency Response ‘the intervention’

In 2007 the Intervention introduced a ban on possession and consumption of alcohol on all land in the NT defined as Aboriginal land by the *Aboriginal Land Rights (NT) Act 1976;* alongside other measures which are described elsewhere [89]. While the Intervention did not interfere directly with existing permits systems, it did with severely limit the powers of the NTLC and the communities themselves, by essentially retaining a ‘veto’ power [29]. Communities that had clubs before 2007 also retained these, with additional restrictions in relation to takeaway alcohol, opening times, and the sale of full-strength beer [27]. In 2010 the Commonwealth reintroduced quasi-community control to Aboriginal communities through Alcohol Management Plans (Commonwealth)^4^ [63]. The instrument gained formal recognition in 2012 through the introduction of the *Stronger Futures in the Northern Territory 2012* (which superseded the *National Territory National Emergency Response Act 2007)* [19]. These Alcohol Management Plans required Federal Ministerial approval; and by late 2015 only one had been approved with seven others rejected [90]. In 2016 there was a shift to Alcohol Action Initiatives, a different policy instrument, with a broader scope than Alcohol Management Plans [19]. We are aware of an evaluation of Alcohol Action Initiatives having been commissioned at the time of writing this paper.

#### Town level

##### Territory-wide changes to opening hours

As part of a package of alcohol control initiatives, recommended by the Martin Report of the Working Party on Drunkenness, in July 1982 takeaway alcohol outlet trading hours were reduced across the NT [37]. Hours were reduced again in 1992 with the introduction of LWA.

##### Localised restrictions

In the 1990s community groups began requesting that the NTLC introduce local liquor licensing restrictions. Stockwell et al. [46] suggest that the provision of LWA funding to community groups and LWA-related changes to the Liquor Act in 1992 supported the ability of these groups to petition for local restrictions. In 1993, take-away alcohol access was limited in Elliott, a small town in the Barkly region, at the request of the Gurungu Council. This was ratified by the NTLC [53, 54]. In August 1995 pressure from ‘Beat the Grog’ campaign led by Julalikari Council Aboriginal Corporation and Anyinginyi Congress Aboriginal Corporation resulted in a trial of alcohol restrictions in Tennant Creek. The evaluation found improvements in public order and health and welfare [57]. In April 1996, all Tennant Creek hotels and takeaway outlets (but not licensed clubs or restaurants) licenses were amended with several restrictions regarding trading hours, takeaway sales, and requirement of food in bars. This included a takeaway alcohol ban on the day welfare payments were made (‘Thirsty Thursday’). In November 1997 the Tennant Creek Town Council requested a review of restrictions as “the efficacy of the restrictions had become the subject of controversy in Tennant Creek” (pg. 3) [58]. Gray et al. [60] found that restrictions remained effective in reducing consumption and related harm. After the 1998 evaluation, although some recommendations were adopted by the NTLC, those which involved extending the current restrictions were not [58]. Instead, the Commission stated that it would conduct a further review in November 2000. This evaluation found that since the end of 1999 the restrictions were no longer as effective in reducing crime, but there continued to be a sustained positive impact on health outcomes [58]. In March 1999 the NTLC imposed restrictions on takeaway trading hours in Katherine and although the four licensees went on to appeal this decision, it was upheld [36]. No evaluation was conducted. In 2002 the NTLC introduced a 12-month restriction trial period in Alice Springs in response to ongoing public debate [66]. From 1 April 2002–31 March 2003 only light beer could be sold prior to 12 pm; there were reduced trading hours, and liquor containers greater than 2 l were removed. These restrictions were evaluated by Crundall and Moon [66]. At the behest of Tangentyere Council and Central Australian Aboriginal Congress, Gray [68] reanalysed Crundall and Moon [66] evaluation, critiquing much of the data and subsequent assertions presented. Crundall [67] responded to the review, which was published with further annotations by Gray. All reports agree that there were decreases in presentations to Alice Springs Emergency Department; admissions to the Sobering Up Shelter; and protective custody orders issued by the police.

##### Liquor supply plans

In 2004, the NT Government commissioned the development of an NT Alcohol Framework, with the associated recommendations were formally adopted in 2005. This was followed by the implementation of Liquor Supply Plans, NT Alcohol Management Plans, and the inception of Public Restricted Area (addressed below). Alice Springs was the first town to experience these measures, with an Alcohol Management Plans and Liquor Supply Plans implemented in the second half of 2006. The Liquor Supply Plan included changes to takeaway trading hours, changes to the volume of wine which could be sold; and changes to volume and times at which cask wine and fortified wines could be sold (for the comprehensive list please see [71]). Following this, several additional measures were introduced, including declaration of the town as a Public Restricted Areas (August 2007), income management (as part of the Intervention in August 2007), and implementation of Alcohol Takeaway Identification cards (June 2008). In Alice Springs (and Katherine) anyone purchasing takeaway alcohol was required to show electronic photo identification for the licensee to check if the individual was subject to a Prohibition Notice or restrictions imposed by a court [73]. The Alcohol Management Plan was evaluated [69], but subsequently, the People’s Alcohol Action Coalition requested a formal critique of this evaluation [70]. A report of all alcohol control measures enacted in Central Australia from 2000 to 2010 found these measures significantly contributed to a reduction in per capita consumption in Central Australia [71].

In July 2006, a Liquor Supply Plan took effect in Tennant Creek. This included the removal of ‘Thirsty Thursday’, with new restrictions on the sale of cask wine and other beverages (for full list see [59]). On 1 Aug 2008, following increases in alcohol-related harms, a second Liquor Supply Plan (called an Alcohol Supply Plan) was introduced, including reductions of takeaway outlet trading hours, further reductions of trading hours for the sale of cask and fortified wine and purchase limits on cask wine, fortified wine and bottles of beers over 750 ml. At this time Tennant Creek town was declared a Public Restricted Area. The evaluation found that while assaults and apprehensions did decline after the introduction of the Liquor Supply Plans and Alcohol Management Plan, they did not fall to what they had been prior to the 2005/06 financial year, when ‘Thirsty Thursday’ was in force [59].

In November 2007 the Katherine Alcohol Management Plan was endorsed by the NTLC, and the Katherine Liquor Supply Plan commenced in January 2008. The Liquor Supply Plan included reduced takeaway outlet trading hours, and purchase limits on cask wine and fortified wine (for full list see [64]). A Public Restricted Areas came into effect on 21 Jan 2008 [64]. Alcohol-related harm did decline for 3–6 months after the introduction of the Liquor Supply Plan, but then subsequently rose again and surpassed pre-Liquor Supply levels [64]. In 2013, the Alcohol Managmeent Plan was revised. From the 3rd quarter of 2013 to mid-2015, both consumption and levels of harm (including alcohol-related offences and public order offenders) declined, but after mid-2015 some harms, notably domestic violence assaults, began to increase, and it was unclear if the harm reduction could be sustained [65].

##### Iterations of uniformed officers at takeaway outlets

Initially called Temporary Beat Locations (TBLs), subsequently renamed Point of Sale Interventions (POSIs) [91], this intervention involved “stationing police outside takeaway outlets to prevent purchases by persons who could not nominate a private address where the liquor would be consumed” [65]. TBLs/POSIs have been in operation on a part-time basis since 2012 in Alice Springs. The stationing of TBLs and POSIs were not legislative interventions, but they did lay the foundation for PALIs, which is a legislative intervention and as such have been included. The substantial cost of placing full salaried police officers to stand outside takeaway outlets, and uncertainties regarding the legalities of this process [7], are considered to have contributed to the inconsistent application of the scheme, with a mix of full and part-time coverage, across Alice Springs, Katherine and Tennant Creek between 2012 and 2018. PALIs are uniformed inspectors, stationed at takeaway alcohol outlets, who seek to prevent the consumption of alcohol in public and restricted areas. PALIs request a form of identification and ask patrons questions regarding their intended drinking location. Purchase will be prevented if a valid (non-restricted) address cannot be provided [10]. The first squad of PALIs graduated in August 2018 and commenced duties in Alice Springs [92]. The 2018–19 NTPFES Annual Report notes 34 PALIs are located in Alice Springs (full coverage was achieved by 3rd Oct 2018), 22 in Katherine (full coverage was achieved by 3rd Jan 2019) and 4 in Tennant Creek (which commenced operations in late Dec 2018) [92]. PALIs have not been evaluated.

#### Individual level

##### Banned drinker register v1 (BDR1)

The first iteration of the BDR was introduced in July 2011 by a Labor government. Individuals who came into contact with the justice system frequently or committed certain offences while intoxicated (e.g., domestic violence) were placed on the BDR1 [76]. Once on the BDR, an individual was prohibited from purchasing, possessing or consuming alcohol for three, six or twelve months [74]. Electronic identification (ID) scanners linked to the Register were in place at all take-away outlets, allowing for identification of banned drinkers and refusal at the point of sale. Anyone wishing to purchase alcohol was required to present their ID, which was then scanned by the computer, with the patron’s ID was compared to the list held on the computer. No record of the customer or their purchase was kept. The instrument went on to become a feature of the 2012 election campaign debate; the Labor party proposed retaining the policy while the Country Liberal party strongly refuted any benefit of the BDR [93]. When the Country Liberals were elected in August 2012 the BDR1 ceased (in a practical sense) immediately and was officially repealed on 1 July. No evaluation was undertaken.

##### Alcohol protection orders (APOs)

Introduced by a Country Liberal government, this policy gave police a discretionary power to issue an APO to any individual charged with an alcohol-related offence associated with a custodial sentence of 6 months or more [34]. Coming into effect on 18 Dec 2013, an APO prohibited the individual from consuming alcohol or entering licensed premises; breaches of an APO could result in up to 3 months of imprisonment [34]. Buckley [74] highlighted that the Act granted police power to breath test people they reasonably believed to have consumed alcohol while subject to an APO and coupled APO enforcement with the policing of takeaway outlets (described above). APOs were repealed in 2017 and were never evaluated.

##### Banned drinker register v2 (BDR2)

The *Alcohol Harm Reduction Act 2017* reintroduced the Banned Drinker Register (BDR2). A Banned Drinker Order (BDO) prohibits the purchase, possession or consumption of alcohol for three, six or twelve months. BDOs can be issued by police, courts, corrections and the BDR Registrar. The Registrar pathway allows individuals to self-refer, be referred by family, or be referred by an authorised person (health professionals, social services and child protection workers). The length of some BDOs can be reduced if the person undergoes voluntary alcohol treatment. In Tennant Creek on 28 February 2018 additional emergency restrictions were introduced in response to an incidence of severe child sexual assault. These restrictions, albeit with some variation between locations as per the NTLC decision [94], remain across the Barkly region. The BDR scanners are used to monitor the additional daily purchase restrictions in the Barkly, despite these restrictions not being linked to the BDR2. A 6-month process evaluation of the BDR2 was released in June 2018 [75], followed by a two-part 12-month evaluation [76, 80] and 24-month evaluation [81]. Relatively few individuals on the BDR escalated their frequency and types of contact with the justice system [76], and post-BDR 51% of banned drinkers had no further alcohol-related contact with the justice system (average 6-month follow-up) [81]. Two qualitative articles explore industry views about the BDR [95] and the impact of the BDR on secondary supply [9]. The BDR2 remains in place.

### Modifying the drinking environment

#### Two kilometre law

In 1983 consumption of alcohol in public within two kilometres of a licensed premises (or on unoccupied private land without the owner’s permission) was prohibited [31]. Like the change in trading hours, the ‘Two Kilometre Law’, was part of the Martin Report of the Working Party on Drunkenness package and was evaluated by the Drug and Alcohol Bureau [38]. O’Connor [40] found drinkers who were visiting from remote communities had largely moved from public drinking locations (such as the Todd River bed) into the town camps^5^ which was considered to have contributed to an increase in alcohol-related violence there. The evaluation found that the law was effective in reducing public drunkenness, but unsurprisingly “while less people are drinking in public, more people are being apprehended for being drunk in public” (pg. 7) [38]. As the law was introduced as part of a package “it is important to note that the impact of the public drinking legislation is difficult to assess in isolation (pg. 3)”. ‘Local dry area alcohol bans’ have been categorised as inherently discriminatory, negatively impacting Aboriginal people who are already at-risk of alcohol-related harms [36]. The law has been discussed in subsequent academic commentaries which address the racialisation of alcohol policy in the NT [30, 34, 41].

#### Regulating strip shows in public bars

In response to an increase in the marketing of strip shows at the Tennant Creek Hotel Anyinginyi Congress Aboriginal Corporation^6^ lobbied the NT Government against the “use of strip shows to sell alcohol in Aboriginal communities [as] one of the latest stages in a long process of colonial exploitation” [42]. In 1989 the Northern Territory Liquor Act was amended to mandate the previously voluntary code of ethics relating to strip shows [42]. The process is described by Boffa et al. [42] but not formally evaluated.

#### Public restricted areas

In September 2006 the *Liquor Act 1978* was amended to allow for the declaration of ‘dry towns’. This intervention was announced as a direct response to growing levels of antisocial behaviour in Alice Springs [36]. Only police, the licensing authority, or a local authority were able to lodge a Public Restricted Areas [34]. The Alice Springs Public Restricted Areas was the first to be implemented on 1st August 2007; and was considered to be “directed primarily against Indigenous people (pg. 98)” [36]. Public Restricted Areas have been considered in evaluations of Liquor Supply Plans and Alcohol Management Plans but never evaluated in their own right. Based on a recommendation from Northern Territory Alcohol Policies and Legislation Review [11] in 2019 all public spaces within urban areas were declared restricted, with the option for exemptions. This essentially subsumed Public Restricted Areas and the 2 Kilometre Law.

### Drink-driving prevention and countermeasures

#### Tightening drink driving regulation

In mid-1992 the NT government passed legislation reducing the maximum blood alcohol content (BAC) for drivers of cars, light trucks, and motorcycle riders from 0.08 to 0.05; although the law was not gazetted until December 1994 [50]. While this was largely driven by a Commonwealth initiative which tied road maintenance funding to the introduction of 0.05 BAC, LWA funds were used to provide complementary mass media campaigns [46]. As noted above, lowering the BAC was considered to have contributed to the success of LWA [45, 46].

### Restrictions on marketing

This overview found no legislated alcohol policies which focused on marketing restrictions.

### Education and persuasion

This overview found no legislated education and persuasion policies, although some funds raised through the LWA levy were allocated to education activities [46].

### Treatment and early intervention

It is unusual to capture treatment within an overview of legislated alcohol policy, as treatment is rarely legislated. However, there are three examples of this within the NT. In addition, some LWA levy funds were allocated to treatment services [46].

#### Alcohol courts

MacKeith et al. [70], Senior et al. [69], and Symons et al. [71] all mention the Alcohol Courts, introduced in 2006 by a Labor government. These courts provided alternative sentencing for people who committed alcohol-related offences and appear to be dependent on alcohol [70] with Senior et al. [69] suggesting they were underutilised. It is unclear when they ceased operation.

#### Substance misuse and referral for treatment (SMART) courts

SMART Courts were implemented by a Labor government on 1 July 2011. These courts provided an option for offenders with a history of serious substance misuse found guilty of committing certain offences to receive a range of alternative sentencing orders which were not punitive and instead emphasised rehabilitation [74]. The intervention was based on the Swift Certain Fair model of response to alcohol and drug-related crime [96] that has been trialled in jurisdictions in the USA and UK [97–99]. SMART courts were repealed by Alcohol Mandatory Treatment Act 2013 (NT) and were never formally evaluated.

#### Alcohol mandatory treatment (AMT)

On 1 July 2013 legislation introduced by a Country Liberal government required individuals who were taken into police custody as a result of intoxication on three occasions in two months or less to receive alcohol treatment under the *Alcohol Mandatory Treatment Act 2013.* Individuals could be mandated to receive a community treatment order (in a residential or community setting); a mandatory residential treatment order for up to three months; or a release or exemption order. This decision was made by the AMT Tribunal. Individuals who were eligible welfare recipients were also subject to an income management order [77]. AMT was openly targeted at chronic drinkers who were publicly intoxicated and unlike SMART Courts was not available to those who committed crimes while intoxicated. Assessment and mandatory treatment services were established in Darwin, Nhulunbuy, Katherine, Tennant Creek and Alice Springs, but services were discontinued in Nhulunbuy in 2014, and Tennant Creek in Jan 2016, although assessments continued through Tennant Creek Hospital [77]. When a Labor government came into power in August 2016 they immediately began ‘winding back’ AMT [77]; on 1 September 2017 AMT was officially repealed through the *Alcohol Harm Reduction Act 2017.* AMT was officially evaluated by PwC’s Indigenous Consulting [77], which found no long-term health or social benefits, with most AMT clients “re-apprehended by NT Police multiple times, entering custody from homelessness and ending up homeless again” (pg. iii). Other researchers [34, 74, 78, 79] have raised several legal and ethical concerns, including using a medical intervention to address social issues; de-facto discrimination against Aboriginal people; opacity regarding the tribunal proceedings; a dearth of legal representation before tribunals; and no right to legal representation for the individuals awaiting assessment (who could be held for up to 96 hours).

## Discussion

As will now be clear to the reader, since 1979 there have been numerous legislated alcohol policies introduced in the NT. In some cases, these have been driven by local community organisations’ advocacy and in other cases by political will. The politicisation of alcohol policies has led to quick turnovers of these policies in the past decade. Indeed, in the lead up to the 2020 NT election the Country Liberal Party included the removal of MUP (as Labor policy) within their campaign, while Territory Alliance (a new party led by a former Country Liberal leader) discussed the reintroduction of AMT. Labor went on to win the election. Generally, NT-wide reforms have been enacted with swift investment in multiple interventions, often in relatively short time periods, likely reflecting the political will of the time. This can make it difficult to evaluate the impacts of single interventions - although not impossible, depending on the timeframes involved, the geographic variations in implementation, and the outcome measures used. For example; the recent MUP evaluation was able to use Darwin, as an urban centre without PALIs, as a quasi-control to demonstrate that MUP made a unique contribution to harm reduction, independent of regionally-specific policies [85]. A similar approach was used by Symons and colleagues who used Darwin as a quasi-control when considering the cumulative impact of alcohol restrictions in Alice Springs from 2000 to 2010 [71].

The NT is a national leader in pricing policies; neither LWA nor MUP have been legislated in any other Australian jurisdiction. This is encouraging because pricing is one of the most effective harm minimisation policy levers [100]. The vast majority of legislation alcohol policies in the NT have focused on regulating the availability of alcohol. This may partly be an artefact of the search perimeters, as alcohol availability policies are much more likely to be legislated than education campaigns or funding for treatment services, but may also reflect the politicisation of alcohol policy in the NT and the requirement for politicians to be seen to be ‘doing something’. Controlling alcohol availability has been shown to reduce alcohol consumption and related harms [100] and certainly some of the NT policies which targeted availability have demonstrated positive outcomes (see Supplementary Material: please note this a descriptive table).

Policies which have focused on the Modifying the Drinking Environment have largely revolved around improving urban amenity (e.g., the 2 km Law, Public Restricted Areas), aiming to shift Aboriginal people’s consumption out of sight [30, 32, 34, 36, 41]. This is also the case for AMT. Prohibiting public drinking results in displacement, demonstrates no evidence of reducing alcohol-related harm [101] and negatively impacts marginalised groups by perpetuating harmful health and social inequities, including systemic racism. Aboriginal people were disproportionately represented in AMT, which showed no evidence of ongoing effectiveness [77], and are also overrepresented on the BDR2 [76, 81]. At this juncture we highlight Secombe and colleagues’ [83] recent findings:

*“Furthermore, in contrast to a misperception held in some sections of the Australian public, this data, like previous studies from the NT, confirms that harmful alcohol use is not solely an Indigenous issue (pg. 7)”*

Aboriginal people are more likely to be abstainers than non-Aboriginal people [102], but those who do drink experience greater harms. Indeed, the alcohol-attributable death rate for Aboriginal people in the NT is 9–10 times higher than the national rate, while the rate for non-Aboriginal people in the NT is twice the national rate [103]. It is essential to understand these harms through the lens of the Social Determinants of Health, which for Aboriginal people includes higher rates of both social disadvantage and marginalisation [104], as a result of ongoing colonisation and discrimination. Internationally, there is consistent evidence that people from lower socioeconomic group experience more alcohol-related harm, despite consuming less or the same as those in higher socioeconomic groups. This is called the alcohol harm paradox [105].

In the case of alcohol policies advocated for by Aboriginal communities and organisations, there has often been a focus on the impact of alcohol on culture and family, sometimes specifically regarding alcohol-related violence against women and children [26, 53, 55]. Although these interventions have involved community organisations and/or local leaders, they are underpinned by legisation. As highlighted by Shakeshaft et al.’s [106] cluster randomised control trial of community alcohol action projects, complementary legislative action is likely to be required to effectively reduce alcohol-related harms in a remote community context, as previously theorised by d’Abbs [31] in his complementary control model. Evaluations of interventions in specific towns or communties have sometimes proved challenging with small numbers in police or health care data, due to the small population size, making it difficult to draw robust statistical conclusions.

Some evaluations note the inability to sustain the initial promising trajectory of outcomes over time. For example, initial decreases in alcohol-related treatments at a health clinic were perceived to be impacted by circumvention of restrictions, with individuals choosing to drink at licensed venues which were not subject to the ‘6 can’ takeaway alcohol limit [53]. In some cases, positive impacts were interfered with by another legislative change, as was seen for LWA when the High Court ruled levies implemented by state and territory governments to be unconstitutional [45]. However, even when the acute impact of restrictions decreases, it is likely that there is an ongoing health benefit for those who suffer from chronic harms, as highlighted by Chikritzhs et al. [45].

### Limitations

This overview drew on published academic and grey literature, and so it is possible that if an intervention did not attract evaluation or academic attention it may not have been captured. We have included grey literature, which has limited or no peer review, thus completeness of the reporting and quality of the evaluations have often not been formally assessed. As this is a historical overview, not a policy analysis, we have not critically assessed the documents identified. We also note some government commissioned evaluation reports may not have been publicly released, and would therefore not have been captured by this overview. For example, Shaw et al. [27] was only publicly released in 2017 once the ABC obtained a copy of the review, after a Freedom of Information request had been refused [107].

## Conclusion

This paper illustrates the patchwork of legislated alcohol policies implemented and repealed over the past forty-two years in the NT. Evaluations of several of these policies have found positive effects; particularly for policies which impact price and availability [26, 45, 46, 60, 86], however several were not evaluated. A number of policies have included an implicit focus on Aboriginal people’s consumption and related harms [34], often without strategies which adequately address the intergenerational effects of colonisation, trauma, and disempowerment. It is clear that alcohol policy is politicised in the NT, as demonstrated by advocacy organisations requests for reviews of evaluations [68, 70] and by the stark policy shifts which have resulted from changes in government. This has been particularly noticeable in the past decade. This focus on the political favourably of policies can draw attention away from the evidence base; with policy decisions based on public discourse rather than effectiveness. Given that the levels of alcohol-related harm in the NT remain the highest in the nation, there is a strong argument to be made for bipartisan support of evidence-based interventions.

## Supplementary Information

**Additional file 1.** Supplementary material: descriptive summary of Northern Territory legislated alcohol policy evaluation reports from 2007 to 2020.

## Data Availability

All data generated or analysed during this study are included in this published article [and its supplementary information files].

## Footnotes

1 Henceforth the term Aboriginal will be used to respectfully refer to Aboriginal and/or Torres Strait Islander people, who are the traditional owners of Australia. This is for brevity only and should not be taken as an indicator of homogeneity between language groups.

2 This overview will mention the NT Liquor Commission, as an independent statutory body. To reduce confusion, it will be referred to as NTLC throughout the paper, however we note the Liquor Commission was renamed the NT Licensing Commission under the *Northern Territory Licensing Commission Act 1999*. In January 2015 the NTLC was abolished by a Country Liberal government and its function transferred to within the NT Government (Director-General of Licensing), thus removing its independence and in essence removing a statutory process for community input. The NTLC was reinstated by a Labor government through the *Liquor Commission Act 2018.*

3 A collective term for Aboriginal people who identify with language groups located on Pitjantjatjara and Ngaanyatjarra land.

4 In the NT there have been two separate policy instruments, both called Alcohol Management Plans, one which was driven by the NT Government and one which was driven by the Commonwealth Government.

5 The term ‘town camp’ is sometimes used to refer to Aboriginal communities situated within or close to town boundaries.

6 Now Anyinginyi Health Aboriginal Corporation.
