# Supplementary material for: A historical overview of legislated alcohol policy in the Northern Territory of Australia: 1979–2021
Source: BMC Public Health. 2021 Oct 23;21:1921. doi: 10.1186/s12889-021-11957-5 (PMC8539741; doi:10.1186/s12889-021-11957-5)
Supplement: Supplementary file 1 — Additional file 1. Supplementary material: descriptive summary of Northern Territory legislated alcohol policy evaluation reports from 2007 to 2020. [file 12889_2021_11957_MOESM1_ESM.pdf]

Supplementary material: descriptive summary of Northern Territory legislated alcohol policy evaluation reports from 2007 to 2020

Policy	Report	Data sources	Impact on consumption	Impact on harms
Groote Eylandt permit scheme	Conigrave K, Proude E, d'Abbs P. Evaluation of the Groote Eylandt and Bickerton Island Alcohol Management System. Darwin: Department of Justice, Northern Territory Government; 2007.	Administrative data<br><br>Key informant interviews (stakeholders and community residents)<br><br>Survey of residents with a post office box in Alyangula<br><br>Licensing Commission records of the process of formation of the Alcohol Management System	Small decrease (9%) in volume of alcohol sold by licensed premises; notable decrease in reduction of heavy beer (20%), partiality offset by an increase in mid-strength beer (11%)	People, particularly women, in the community reported feeling safer.<br><br>Police data also demonstrates a 67% decrease in aggravated assaults (lowest in four years), 86% reduction in house break-ins, 23% decrease in number of adults admitted to correctional centres from Groote Eylandt and Bickerton Island (also lowest in four years).<br><br>Some concerns were raised regarding community members leaving Groote Eylandt to access alcohol, although the responses to this were mixed.
Alice Springs Liquor Supply Plan and Alcohol Management Plan (LSP / AMP)	Senior K, Chenhall R, Ivory B, Stevenson C. Moving beyond the restrictions: the evaluation of the Alice Springs Alcohol Management Plan. 2009.	Administrative data<br><br>Key informant interviews<br><br>Participation in and observation of relevant town meetings<br><br>Review of the activities of alcohol related interest groups<br><br>A description of services involved in alcohol misuse	Adjusted for seasonal variability total alcohol sales show a significant downward trend from Mar 2005 – Dec 2008 ($p<0.001$)<br><br>After September 2006 (LSP / AMP introduction) downwards trend was significantly lower than the trend before September 2006 ($p<0.001$).<br><br>LSP / AMP intervention had an effect on alcohol sales in addition to the existing downward trend	Alcohol related hospital separations as a proportion of total separations reduced from 16.2% prior to the LSP / AMP introduction to 14.1% in the year following the introduction ($p < 0.000$).<br><br>Significant reduction in the proportion of total hospital separations which were due to assault: 7.0% prior, 6.4% 12-months post ($p=0.01$); 5.7% 24-months post ($p = 0.007$).<br><br>Absolute number of police recorded assaults did not change significantly, however the proportion of serious assaults decreased in the year after LSP / AMP ($p=0.004$).<br><br>Total property offences rose significantly in both 2007 and 2008 ($p < 0.0001$), particularly the

Policy	Report	Data sources	Impact on consumption	Impact on harms
		Outlines of governance structures maintaining the Alcohol Management Plans<br><br>A survey of community attitudes and receptivity to LSP / AMP (in Alice Springs; and then separately in town camps)<br><br>Formal submissions to the evaluation		proportion of commercial break-ins. Important to note the overlap with introduction of income management.<br><br>Extensive discussion regarding community perceptions of the restrictions, including systemic and overt racism.
Alice Springs Liquor Supply Plan and Alcohol Management Plan (LSP / AMP)<br><br>Re-evaluation: prepared for the Alice Springs People's Alcohol Action Coalition	MacKeith S, Gray D, Chikritzhs T. Review of: Moving beyond the restrictions: The evaluation of the Alice Springs Alcohol Management Plan. A report prepared for the Alice Springs People's Alcohol Action Coalition Perth: National Drug Research Institute: Curtin University of Technology; 2009.	A review of data, analysis, and assertions presented in Senior et al. (2009)	Senior et al. (2009) caveat the above calculations as under-estimation of consumption.<br><br>MacKeith et al. (2009) state it is more likely to be an over-estimation as per capita should be calculated as sales from all outlets in the region, using estimated residential population of region plus estimate of tourist numbers - as per Gray & Chikritzhs (2000).	Reasons for review: scope of report does not address terms of reference; inadequate and uncritical review of literature; dearth of comparison sites; failure to consider several key indicators; incorrect analysis methods; no separation of data by Aboriginality, and a "general lack of rigour and apparent lack of expertise in the field [which] has led to… a set of inadequate, if not potentially harmful, recommendations (pg. 11)."<br><br>Overall, this re-evaluation asserts that the magnitude of reduction of harms (particularly regarding lack of inclusion of grievous bodily harm offences, road traffic accidents, alcohol-attributable injuries in hospital separations) and consumption were underestimated by Senior et al. (2009) and presents alternate recommendations.

Policy	Report	Data sources	Impact on consumption	Impact on harms
Tennant Creek Liquor Supply Plan and Alcohol Management Plan (LSP / AMP)	d'Abbs P, Ivory B, Senior K, Cunningham T, Fitz J. Managing alcohol in Tennant Creek, Northern Territory: an evaluation of the Tennant Creek Alcohol Management Plan and related measures to reduce alcohol related problems. Darwin: Menzies School of Health Research; 2010.	Administrative data	2006-07 (after Thirsty Thursday – TT - removed) total alcohol supply to outlets increased by 7.5%<br><br>2007-08 total alcohol supply remained stable<br><br>2008-09 (after AMP/LSP) total sales fell by 4.4%*<br><br>*this figure was provisional	Aboriginal alcohol-related presentations at the Tennant Creek Hospital Emergency Department increased after TT was removed; 56% increase in 2007-08 and a further 61% increase in 2007-08. After Aug 2008 (AMP/LSP) the upward trend was reversed but, there was still a 61% total increase compared to 2006-07.<br><br>Alcohol-related assaults increased by 3.8% directly after TT was removed and increased by a further 24.1%. Although they reduced by 24.8% after AMP/LSP, there was no net improvement from 2006-07.<br><br>Following the AMP/LSP numbers of public order incidents decreased by 27.1%, representing a decline of 25% from TT levels. AMP/LSP had clear, positive outcome on this indicator.
Katherine Liquor Supply Plan and Alcohol Management Plan (LSP / AMP)	d'Abbs P, McMahon R, Cunningham T, Fitz J. An evaluation of the Katherine Alcohol Management Plan and Liquor Supply Plan Darwin: Northern Territory Department of Justice 2010.	Administrative data<br><br>Telephone survey	In three calendar years preceding LSP/AMP (2005 – 2007) total volume of alcohol supplied in Katherine town remained stable, although supply to outlets in surrounding area increased.<br><br>Following LSP/AMP (2008) total alcohol supply in and around Katherine declined by 12.2%, although this was partly offset by a 4.2% increase in 2009.	All administrative data examined (emergency department presentations for alcohol-related disorders; alcohol-related and injury hospital separations; assaults; disturbances and anti-social behaviour; and public drunkenness) demonstrated similar trends, with an immediate decrease followed by an upwards trend that surpassed pre-LSP/AMP levels.<br><br>The most notable being an 8.5% total increase in apprehensions for public drunkenness, 16.1% total increase in alcohol-related hospital separations and 32% total increase in alcohol-related assaults.

Policy	Report	Data sources	Impact on consumption	Impact on harms
Gove Peninsula permit scheme	d'Abbs P, Shaw G, Rigby H, Cunningham T, Fitz J. An evaluation of the Gove Peninsula Alcohol Management System. Darwin: Menzies School of Health Research: A report prepared for the Northern Territory Department of Justice; 2011.	Administrative data<br><br>Stakeholder interviews<br><br>Street survey	Total volume of alcohol supplied to Nhulunbuy outlets decreased by 22.3% (April 2008 – March 2009) compared to previous 12 months<br><br>The following 12 months (April 2009 – March 2010) total supply decreased by a further 12.3%.<br><br>Permit system appears to have contributed to a sustained downwards trend (which had begun at the end of 2006 with a voluntary cessation of sale of cask wine by some outlets)	Aboriginal presentations to Gove Hospital Emergency Department coded as 'mental and behavioural disorders due to alcohol' decreased by 22% in 12 months following permit system. The next 12 months this number decreased by another 50%. Aboriginal hospital separations for this code also fell by 35.8% and then a further 7.1%<br><br>Hospital separations for injury in Aboriginal patients decreased by 9.3% and non-Aboriginal separations declined by 13.7% in the 12 months following the permit system<br><br>Trends in disturbances and anti-social behaviour also declined, however this decline had begun prior to the introduction of the permit system.
Remote Community Clubs	Shaw G, Brady M, d'Abbs P. Managing Alcohol Consumption: A review on licensed clubs in remote Indigenous communities in the NT. Canberra: Bowchung Pty Ltd; 2015.	Administrative data<br><br>Surveys with residents and services in the 8 communities with clubs (Beswick, Gunbalanya, Milikapiti, Wurrumiyanga, Peppimenarti, Pirlagimpi, Kalkarindji & Wurankuwu)	Since the Intervention (2007) introduced restrictions on club trading hours, ban on full strength beer and takeaway (in conjunction with income management) 5 clubs recorded significant decline in wholesale supply; 2 clubs (the smallest) had insufficient data to examine trends; and 1 club demonstrated no change until 2009, after which it slightly declined. Gaps in records for this club preclude definitive conclusions.	Prior to and at the time of the Intervention (2007) alcohol-related harms were much higher in communities with clubs, however since then harms in club communities have reduced while harms in NT as a whole and other communities have increased.<br><br>Hospital separations indicate communities with clubs have a slightly lower rate of alcohol-related separations than those without clubs.<br><br>Shaw et al. (2015) conclude that communities with clubs do not experience a marked difference in alcohol-related harms compared to other communities, however adherence to shortened hours and a ban on full-strength beer is important.

Policy	Report	Data sources	Impact on consumption	Impact on harms
Katherine Revised Liquor Supply Plan and Alcohol Management Plan (LSP / AMP)	d'Abbs P, Whitty M. Implementation and outcomes of the revised Katherine Alcohol Management Plan: an evaluation: A report for the Katherine Region Action Group. Darwin: Menzies School of Health Research; 2016.	Administrative data<br><br>Semi structured interviews with stakeholders regarding Katherine Region Action Group's role and effectiveness.	From 2006 until late 2013, apparent consumption remained steady – though subject to a recurring seasonal cycle<br><br>From 3rd quarter 2013 (LSP/AMP) the trend has been downwards; wholesale supply in first quarter of 2015 was 24.9% lower than in corresponding quarter of 2004 – however it considered too early to draw concrete conclusions from this trend.	In Katherine in 2015, 76.6% of assaults were associated with alcohol, and 72.4% with domestic violence.<br><br>Assaults in Katherine increased between 2006 and 3rd quarter 2013, this trend shifts to downwards until 1st quarter 2015 when it began to increase again, particularly for domestic violence assaults.<br><br>d'Abbs & Whitty (2016) note it is too early to tell if reduction in harm could be sustained.
Liquor Permit Schemes	d'Abbs P, Crundall I. Review of Liquor Permit schemes under the NT Liquor Act: Final Report. Darwin: Menzies School of Health Research; 2016.	Interviews with community members and local staff, including police	Although permits may been seen as a method of encouraging moderate consumption most purchasing entitlements are in excess of NHMRC consumption guidelines for minimising alcohol-related harms, and therefore do not promote low-risk consumption.	74 (77%) of NT Aboriginal communities do not have a permit scheme; 14 have exemption schemes, which allow for staff living and working in the community be exempt from 'dry' conditions; and 8 have permit-based alcohol management systems (Maningrida, Wurrumiyanga, Milikapiti, Pirlangimpi, Alyangula, Nhulunbuy, Yirrkala, Gunyangara).<br><br>The liquor schemes were considered to provide benefits to communities and to be generally well accepted. The review identified some issues and inconsistencies with the application of graduated permit levels, what evidence Liquor Permit Committees should consider admissible and inadmissible, and the need to maintain a balance between community control and centralised bureaucratic management. Secondary supply remains an issue, particularly in the context of humbug (pressure to share).

Policy	Report	Data sources	Impact on consumption	Impact on harms
AMT	PwC Indigenous Consulting. Evaluation of the Alcohol Mandatory Treatment Program. Northern Territory Department of Health 2017.	Client outcomes compared to similar cohort who did not receive AMT<br><br>Linked administrative data<br><br>AMT process and implementation data<br><br>Case studies<br><br>Expenditure on program	No consumption data presented.<br><br>Logically, AMT only affected a small percentage of NT residents and is unlikely to have impacted wholesale supply data.	Although some participants reported short term improvements in health as a result of access to services provided with AMT (ie. dental care), no ongoing health benefits were identified.<br><br>The majority of participants cycled in and out of AMT with most "re-apprehended by NT Police multiple times, entering custody from homelessness and ending up homeless again" (p. iii).<br><br>There was a high rate of absconding, some individuals absconded up to four times while serving one treatment order
BDR 6-month process evaluation	Smith J, Adamson E. Process evaluation of the Banned Drinker Register in the Northern Territory. Darwin: Menzies School of Health Research; 2018.	Administrative data<br><br>BDR process data<br><br>Key informant interviews with policymakers, frontline staff and industry representatives	No consumption data presented.	At the end of Feb 2018 2,905 were on the BDR (64% for a police ban, 28% for a court ban, 4% for a parole ban, and 4% for a BDR Registrar ban).<br><br>87% of people on the BDR were Aboriginal, 73% were males.
BDR 1-year evaluation	Smith J. Twelve-month Evaluation of the Banned Drinker Register in the Northern Territory: Part 1 - Description Analysis of Administrative Data. Darwin: Menzies School of Health Research; 2018.	Part 1: Administrative data<br><br>Part 2: Semi structured stakeholder interviews	Wholesale data is presented for NT as a whole and each region in graphs. Trend lines appear to demonstrate stability in NT as a whole, with a mix of slight increase and decreases across regions. No reference to alcohol wholesale supply is made within the text.	There was no immediate impact on the frequency of alcohol-related events, however there was a statistically significant gradual decline in the rate of individual's alcohol-related events once they were on the BDR. Relatively few individuals on the BDR escalated their frequency and types of contact with the justice system.<br><br>Aboriginal people were significantly overrepresented on the BDR (87.9%) - likely reflecting the over-representation of Aboriginal

Policy	Report	Data sources	Impact on consumption	Impact on harms
	Smith JA, Adamson E, Clifford S, Wallace T. Twelve-month evaluation of the Banned Drinker Register in the Northern Territory: Part 2 – A qualitative analysis of selected stakeholder perspectives. Darwin: Menzies School of Health Research; 2019.			people in the justice system: 64% of bans were police-issued and 26% were court-issued.<br><br>Uptake of referral pathways outside of the justice system was low - 10% of people referred by sources other than the police or courts.<br><br>Use of therapeutic services among people on the BDR was low – only 7% had commenced treatment in the first 12 months of the BDR.
MUP 1-year evaluation	Coomber K, Miller P, Taylor N, Livingston M, Smith J, Buykx P, et al. Investigating the introduction of the alcohol minimum unit price in the Northern Territory: Final Report. Deakin University, Geelong Australia: Prepared for the Northern Territory Department of Health; 2020.	Administrative data<br><br>Population telephone survey<br><br>Key informant interviews<br><br>Price monitoring<br><br>Monitoring sales of substitution commodities	Greater Darwin region: cask wine and cider wholesale supply per capita declined<br><br>Alice Springs: steep decrease in overall wholesale supply per capita of alcohol<br><br>Katherine: significant decrease in the wholesale supply of cask wine and bottled wine per capita but an increase in light beer<br><br>Tennant Creek: a significant gradual increase in the supply of mid strength beer per capita	Greater Darwin region<br>• significant decreases in the rate of alcohol-related assault offences, protective custody episodes, and alcohol-related ambulance attendances<br>• some decline in the rate of alcohol-related hospital admissions and although there was an initial increase in the rate of other substance use hospital admissions, this was followed by a gradual decline<br>• significant slope decreases in the rate of assault-related hospital admissions<br>• no significant change to the volume of alcohol wholesale supply to nightlife venues

Policy	Report	Data sources	Impact on consumption	Impact on harms
				Alice Springs<br>• significant declines in the rate of police recorded alcohol-related assault offences, protective custody episodes, alcohol-related ambulance attendances, alcohol-related ED presentations, alcohol-related hospital admissions, and Sobering Up Shelter admission<br><br>Katherine<br>• declines in the rate of alcohol-related assault offences, alcohol-related ambulance attendances, and alcohol-related hospital admissions<br><br>Tennant Creek<br>• a decrease in the rate of alcohol-related ambulance attendances, and alcohol-related ED presentations.
BDR 24-month evaluation	Ernst & Young. Medium Term (24 months) Outcomes Evaluation of the Banned Drinker Register. Darwin: Northern Territory Department of Health; 2020.	Review of relevant policy documentation<br><br>Administrative data<br><br>Key stakeholder interviews<br><br>Two online surveys of Therapeutic Service Providers and Licensed Premises Managers	Comparative to 2017, 2018 wholesale alcohol supply data demonstrates a 2% decrease in estimated per capita consumption (11.55 litres per person to 11.27 litres per person)	Between February and August 2019 there was an average of 3819 people on the BDR; 65% via police ban, 26% via court ban, 6% via the BDR Registrar and 3% via corrections (parole). Aboriginal people are overrepresented on the BDR (84%).<br><br>The introduction of PALIs was associated with a substantial downwards trend in alcohol-related assault offences, assault apprehensive, and protective custody episodes; however, the report notes that the impact of the BDR may be cumulative as banned drinkers enter the register incrementally, and they attribute the decreasing

Policy	Report	Data sources	Impact on consumption	Impact on harms
				trends to the range of alcohol initiatives introduced (BDR, PALIs, MUP).<br><br>Post-BDR 51% of banned drinkers had no further alcohol-related contact with the justice system (average 6-month follow-up).
